# ‘*The greatest feeling you get, knowing you have made a big difference’:* survey findings on the motivation and experiences of trained volunteer doulas in England

**DOI:** 10.1186/s12884-016-1086-6

**Published:** 2016-09-29

**Authors:** Helen Spiby, Jenny Mcleish, Josephine Green, Zoe Darwin

**Affiliations:** 1 Division of Midwifery, Faculty of Medicine and Health Sciences, University of Nottingham, 12th Floor, Tower Building, Nottingham, NG7 2RD UK; 2 Policy Research Unit for Maternal Health and Care, National Perinatal Epidemiology Unit, University of Oxford, Oxford, OX3 7LF UK; 3 Department of Health Sciences, University of York, Heslington, York, YO10 5DD UK; 4 Midwifery, Social Work and Counselling & Psychotherapy, School of Healthcare, University of Leeds, Leeds, LS2 9JT UK

**Keywords:** Trained volunteer doulas, Social disadvantage, Doulas’ experiences, Maternity, Peer support

## Abstract

**Background:**

Support from a doula is known to have physical and emotional benefits for mothers, but there is little evidence about the experiences of volunteer doulas. This research aimed to understand the motivation and experiences of volunteer doulas who have been trained to support women during pregnancy, birth and the postnatal period.

**Methods:**

A postal questionnaire survey was sent to volunteer doulas at five volunteer doula projects working in low-income areas in England. Quantitative and qualitative data were analysed in parallel using summary statistics and content analysis respectively.

**Results:**

Eighty-nine volunteer doulas (response rate 34.5 %) from diverse backgrounds responded to the survey. Major motivators for volunteering included a desire to help others and, to a lesser extent, factors related to future employment. Most reported that the training was effective preparation for their role. They continued volunteering because they derived satisfaction from the doula role, and valued its social aspects. Their confidence, skills, employability and social connectedness had all increased, but many found the ending of the doula-mother relationship challenging. For a minority, negative aspects of their experience included time waiting to be allocated women to support and dissatisfaction with the way the doula service was run.

**Discussion and conclusions:**

Most respondents found the experience rewarding. To maintain doulas’ motivation as volunteers, services should: ensure doulas can start supporting women as soon as possible after completing the training; consider the merits of more flexible endings to the support relationship; offer opportunities for ongoing mutual support with other doulas, and ensure active support from service staff for volunteers.

## Background

A ‘doula’ is a woman who provides social, emotional and practical support to other women during pregnancy, birth and the postnatal period. She is not a health professional and does not provide any clinical care [1, 2]. A critical integrative review of international studies of doula support (usually in highly medicalised birth environments) indicates that support from a trained or professional doula is associated with a range of obstetric and psychosocial benefits for mothers and babies: shorter labours; less likelihood of having an instrumental or caesarean delivery, or an epidural or augmentation of labour; increased likelihood of breastfeeding; more positive expectations of birth, more positive mother-baby interactions after birth, and a greater sense of achievement and self-worth [2]. Mothers and babies appear to be most likely to benefit where the doula has established a relationship with the mother in the antenatal period [3].

Most research has focused on the impact of doula support for mothers and much less is known about the motivation and experiences of the doulas, particularly when the doula is a volunteer. There is limited survey evidence on paid doulas from North America and differences in maternity systems preclude direct comparison with the UK. However, for doulas in the USA, the biggest sources of satisfaction were supporting and empowering mothers and helping mothers to have a positive birth experience. The biggest challenges were the lack of support from clinicians and the difficulty of balancing doula work with other jobs [4]. In a Canadian survey, doulas’ key motivations were the desire to support women in childbirth, personal interest, and a wish to share their own positive birth experience with others [5]. The biggest challenge was the stress of being ‘on call’. Doulas in the Canadian survey were much more likely than those in the USA survey to say that they found it very satisfying to support women (97.1 % vs 49 %) and to help women in childbirth (89.6 % vs. 48.2 %). An ethnographic study on the experiences of four paid doulas found their motivation to be a sense of ‘being called’ and a desire to prevent women having negative postpartum experiences [6].

The evidence on unpaid volunteer doulas’ motivations and experiences is even more limited than that of paid doulas. Focus groups with 14 volunteer doulas in the USA (most of whom hoped to become paid doulas) found that their main motivations were a desire to help others, to establish a practice as an employed doula, and to have a route into nursing or midwifery. Key challenges were a lack of clear boundaries, women’s and their partners’ poor understanding of the role, and the clients’ complex socio-economic issues [7].

None of this evidence on doula experiences comes from the UK, where the maternity services context is very different. Antenatal, intrapartum and postnatal care is provided by the National Health Service and is free at the point of delivery, although women with some types of immigration status may be retrospectively charged for their care. Midwives are usually the lead maternity professional for ‘low risk’ pregnancies; the principles of care include maternal choice and continuity of care from a known midwife in the antenatal and postnatal periods [8].

There is, however, an on-going shortage of midwives in the UK such that in some hospitals, the ratio of midwives to mothers is below the level considered safe [9]. A national survey of mothers’ experiences found that two thirds of mothers did not see the same midwife at each visit antenatally and three quarters did not see the same midwife postnatally [10]. A quarter of mothers had been left alone by doctors or midwives during labour at a time when it worried them [10]. It has been suggested that the shortage of midwives has led to a recent increase in UK mothers hiring private doulas to support them during birth [11]. There has also been a move to provide free volunteer doula support for disadvantaged women, who are disproportionately likely to access maternity care late and to experience poor outcomes [12–14]. There is some evidence about UK volunteer doula experiences in an evaluation report of a service offering both volunteer doula support and volunteer peer support (without birth support), which used interviews and focus groups to gather the experiences of an unspecified number of volunteers. The doulas were not distinguished from the non-doulas in the findings but the volunteers in general were motivated by wanting to help others, sometimes because of their own poor maternity experiences. They reported becoming more confident and being proud of the qualification gained through the training; some planned to pursue a career in health or social care although it was not clear whether this had been part of their initial motivation [15].

This paper reports the experiences of volunteer doulas, including motivations for becoming and continuing as a volunteer doula; experiences of the role in working with women, doula services and healthcare professionals and impacts for doulas on their health and for their family. This was part of a larger study [16]; other aspects were reported elsewhere including setting up and sustaining volunteer doula services [17]; and women’s experiences of doula services [18]. The research was carried out at five volunteer doula services in the UK that work in low-income communities to provide a doula service for disadvantaged women. The research team was independent of the doula services.

### The research sites

The five research sites comprised the UK’s first volunteer doula project, established in 2005, and four roll-out sites that were fully operational by 2011. At each site, the doula service recruited potential volunteers from the community through local advertising, and assessed their suitability by an interview. If accepted, the volunteer took part in training accredited by the Open College Network, which covered topics such as birth, breastfeeding, child protection, domestic abuse, cultural diversity and communication skills. Once trained, the volunteer doula would be ‘matched’ by service staff to a woman seeking support, according to her personality, background, location and availability. The doula was expected to provide support through fortnightly visits (weekly in the month before birth) from around the sixth month of pregnancy until 6–12 weeks after birth, and to support the mother at birth if requested. The latter required doulas to be on call for at least a two-week period. The doula role included emotional support, information, and support in accessing other health and social services, but not clinical care. Doulas were reimbursed by the services for expenses such as travel costs, and usually received on-going supervision and support from paid service staff. At some sites, the funding for the doula service was in part explicitly linked to improving education or employment opportunities for the volunteer doulas, because the service was in an area with high unemployment and few employment opportunities for women with limited qualifications or experience.

The women supported by the volunteer doula services were typically referred by health or social care professionals, or could self-refer. Two of the services targeted women from Black and Minority Ethnic (BME) communities and a third served an area with a largely BME population. The most common reasons for women to use the service were that they were isolated and unsupported, had physical or mental health problems, had challenging social circumstances, had particular fears about giving birth, including negative previous experiences of childbearing.

## Methods

### Development of data collection tools

The study took a realistic evaluation perspective, in recognition of the complex intervention being investigated in a real-life setting [19]. Realist evaluation involves hypothesising how an intervention (volunteer doula support) works and under which circumstances. This can be facilitated by generating Context, Mechanism and Outcome configurations for testing through data collection. Hypotheses were developed from a literature review and from gathering information including beliefs about the volunteer doula role held by key informants (managers and project workers, doulas and service users). Managers and project workers in all sites contributed through interviews. In the original site, the views of eleven doulas were gathered through two focus groups. Doulas were selected and invited by the service staff to represent a range of circumstances and experience, and provided written consent. These focus group discussions informed the development of the questionnaires for doulas, enabling content validity. Doulas who participated went on to form a reference panel who tested and commented on the acceptability and clarity of questionnaire booklets as they were developed; their feedback was incorporated supporting face validity.

### Procedure

Two postal questionnaires were developed, including a mixture of closed response questions where respondents could ‘tick all that apply’ and open text questions. One was for volunteer doulas who had supported at least one woman; the questionnaire explored training to be a doula, becoming involved with the doula service, the doula role, workload, matching women and doulas, barriers to and challenges in supporting women, support for doulas, how the doula service fits with other services, the impact of the doula role on women and doulas, ending the relationship with the woman, and stopping volunteering. The second questionnaire was for doulas who had trained but not (yet) supported a woman and was a modified version of the first, omitting sections that were not applicable and exploring why no women had been supported. Both questionnaires were developed specifically for this research and included questions collecting demographic information. The doula reference panel and advisory group provided guidance during the iterative development of the questionnaires. Data collection took place February–May 2013.

Service staff in the five doula services were asked to send questionnaires to all doulas who had completed training, thus maintaining doulas’ confidentiality. Each questionnaire had a covering letter from the research team that explained the independence of the research team from the doula service and emphasised that participation in the study was voluntary. Completed questionnaires were returned directly to the research team in prepaid envelopes. This initial mailing was followed by a postcard three weeks later thanking those who had already completed questionnaires and inviting completion from those who had not yet done so.

### Data analysis

The quantitative data (analysed using Statistical Product and Service Solutions version 20 database (SPSS IBM Corp [20])) and responses to open questions were analysed in parallel. The percentages reported are based on all valid responses to that question. The open-text comments were analysed using content analysis [21]. They were tabulated using an Excel spread sheet to show horizontally all of an individual’s responses to the questions and vertically all of the responses received to any question; this facilitated coding of themes identified in the responses on a question-by-question basis, identification of disconfirming responses and the exploration of linked patterns between questions. Analyses of the numerical data and open-ended text responses were integrated both according to questions and themes. For more details, see [16].

## Results

### Participants

Postal questionnaires were sent to 258 doulas; nine doulas were not included as the doula service staff deemed it inappropriate (e.g. recent bereavement for the doula or a client). Eighty-nine valid questionnaires were received, a response rate of 34.5 %. 71 of the 89 doulas who responded had experience of supporting women one-to-one (the range was 1–19 women per doula). The other 18/89 had trained as doulas but had not supported a woman: five had left volunteering after training, six were awaiting their first client and seven had been unable to support a woman because of personal reasons. The responses of these 18 are included in the sections below on motivation, training, and understanding the doula role. Table 1 shows the doulas’ sample characteristics. Forty doulas reported that they were currently volunteering: 19 of these (47.5 %) were also in paid employment and 4 (10 %) were students.

**Table 1:** Doulas’ sample characteristics (*N* = 89)

	Number	Percentage
Age		
< 30	20	23 %
30–39	42	47 %
40 and over	27	30 %
Age of leaving full time education^a^		
16 or younger	33	37.5 %
17–19	28	32 %
20+	27	30.5 %
Ethnicity		
White	71	80 %
Asian/British Asian	11	12 %
Black/Black British	5	6 %
‘Mixed’	1	1 %
‘Other’	1	1 %

Footnote: ^a^Age of leaving fulltime education numbers total 88, due to missing data

### Motivation for becoming a doula

Participants were provided with a list of possible personal and work-related motivations for training as a doula and asked to indicate for each whether it was ‘not important’, ‘somewhat important’ or ‘very important’. When responses for ‘very important’ and ‘somewhat important’ are combined, the most important personal reasons endorsed were wanting to support women in pregnancy and during childbirth (97 %, *n* = 85/88), and wanting to work with socially disadvantaged women (83 %, *n* = 74/89). This fitted with respondents’ views on who should be prioritized to receive the service if prioritization were necessary. The possible options were ‘women with complex needs’, ‘women with no partner or husband’, ‘women with no support locally’ and ‘women who actively want the service’. The most frequent response was ‘women with no support locally,’ 47 % (*n* = 33/70) and ‘women with complex needs’ 37 % (*n* = 26/70) (some gave more than one response to this question). For work-related reasons, 76 % of respondents (*n* = 68/89) wanted to use existing skills but a fairly high proportion of the volunteers had seen becoming a doula as a way to explore a career in health or social care (67 %, *n* = 58/87), to improve possibilities for further training (55 %, *n* = 47/86) or gaining employment (54 %, *n* = 47/84).

### Motivation for staying involved

Participants were provided with a list of possible personal reasons for remaining involved after training with possible options of ‘not important’, somewhat important’ and ‘very important’. When ‘very important’ and ‘somewhat important’ were combined, almost all respondents indicated their enjoyment of supporting women during pregnancy and birth (99 %, *n* = 69/70) and their belief they were making a valuable contribution (97 %, *n* = 68/70): ‘*The greatest feeling you get knowing you have made a big difference in a person’s/family’s life*’ [Doula 061]. For a high proportion it was important that being a doula gave them an identity beyond their existing role as a mother or worker (84 %, *n* = 59/70), or that they enjoyed the gratitude and appreciation from clients (84 %, *n* = 59/70). The social aspects of the role were also rated very highly: 94 % (*n* = 65/69) said that working with other doulas and the service staff was important, and 91 % (*n* = 64/70) said it was important to them to be part of a team.

Seventeen doulas who had supported women had subsequently stopped volunteering. Most cited personal reasons such as family commitments, studying, or having moved away from the area. However, at one site, where there had been some staffing instability, the reasons given related to dissatisfaction with the attitudes of and support from service staff e.g. *‘unprofessional’* [Doula 051]; *‘felt uncomfortable in office and meetings’* [Doula 052]. All respondents were asked what would encourage or what would have encouraged them to continue volunteering. Fewer than half said that any factor was important other than fewer personal commitments (71 %, *n* = 62/87), better recognition by health services (58 %, *n* = 49/85) or more contact with other doulas (52 %, *n* = 44/84). When asked whether doulas should be paid and women still receive a free service, 35 % (*n* = 24/69) of respondents thought that doulas should be paid, and just under half (49 % *n* = 41/84) of the doulas said that payment would encourage (or would have encouraged) them to continue as a doula.

### Views of training

Doulas rated the most important outcomes of training as increased knowledge (99 %, *n* = 87/88), a sense of achievement (98 %, *n* = 86/88), making friends (94 %, *n* = 83/88) and increased confidence (90 %, *n* = 79/88). Approximately three quarters of the respondents rated gaining a qualification (80 %, *n* = 70/88) and help to get future training or employment (77 %, *n* = 68/88) as ‘important’ or ‘very important’ outcomes of the training. Asked whether they had struggled with anything in the doula training, 68 % (*n* = 58/85) said they did not struggle with anything, but 23.5 % (*n* = 20/85) had difficulties finding time for the training. Doulas were asked to rate a number of factors for their importance in encouraging new volunteers to stay with the doula service. Fifty-eight percent of respondents indicated that ‘not waiting too long before supporting a woman’ was very important; of the options provided, this was the only response that was considered very important by over half of the respondents.

### Understanding the doula role

#### Timing of support

To understand which aspect of their support the doulas thought was most important, they were asked ‘If the service could only provide support at one stage, which should it be?’. Respondents were asked to indicate one only of the following options: ‘support before the birth’, ‘support during labour/birth’ or ‘support after the birth’. The most common answer was ‘before birth’ (40 %, *n* = 35/88), with doulas arguing that this is when the groundwork for everything else is laid, closely followed by ‘birth’ (35 %, *n* = 31/88), on the grounds that birth is what women fear most, especially if they would be alone. Those selecting ‘after birth’ (25 %, *n* = 22/88) usually said that this is when it is hardest to cope with a new role. Just over half of doulas (55 %, *n* = 37/67) thought that their relationship with the mother was not affected by when they first met, with 13 % (*n* = 9/67) saying it *was* affected by the timing, and 31 % (*n* = 21/67) saying that it depended on the women’s needs.

### Is the relationship about friendship?

Doulas’ views on the extent to which their role was about friendship were very mixed. The doula training emphasised the time-limited nature of the relationship. The services’ policies reinforced the ‘professional’ nature of the role by discouraging the volunteers from revealing personal information about themselves and not allowing contact after the end of the period of support. However, there were some doulas who thought friendship was a small (43 %, 38/88) or big part (36 %, 32/88) of their role, and only a minority thought that it was not part of their role (20 %, 18/88). These widely divergent views were expanded on in open text comments, which indicated that some doulas distinguished (unnecessary) friendship from (necessary) friendliness:

*‘It’s about being caring and friendly but not being a friend.’* [Doula 077].

Some doulas saw friendship within boundaries as an important mechanism for building trust and rapport:

*‘I think the lady needs to feel like you are a friend to trust you. The doula needs to explain role fully though or the lady could really think you are a ‘real’ friend’* [Doula 039].

A third group saw it as a spontaneous human reaction:

*‘You do befriend women as you spend time getting to know them, their needs, their family, culture and it just naturally happens*’ [Doula 049].

### Do doulas need to have children?

The doulas had varied views on whether it was important that a doula should have had a child or parenting experience herself. A third of respondents (34 %, *n* = 30/89) said it was ‘not important’, 44 % (*n* = 39/89) ‘quite important’ and 22 % (*n* = 20/89) ‘very important’. Those who thought it was not important emphasised the professionalism of the role achieved through training (‘*Be[ing] qualified and properly trained is enough to conduct and support a mother’* [Doula 047]). Those who thought it was important to have children said either that it gave them more empathy and thus made them more effective *(‘I feel there are experiences that you cannot learn and you are able to empathise-relate to the woman better’* [Doula 003]); or that it gave them credibility from the mother’s point of view ‘*Knowledge, empathy and professionalism matter most but the mothers I worked with were reassured that I had also given birth so knew what they were experiencing* [Doula 051]’.

### Working with health and social care professionals

Two thirds of respondents (67 %, *n* = 43/64) said they got on well with midwives ‘most of the time’ and almost all of the rest (31 %, *n* = 20/64) said they got on well ‘some of the time’; one doula answered ‘not well at all’. The questionnaire did not seek to distinguish between hospital and community midwives. A small number referred to problems when the service was new and poorly understood by midwives. Some explained that some midwives were apparently uncomfortable with having a doula present at birth: ‘*feeling threatened by our role’* [Doula 003]; ‘*[the midwives are] not sure why I’m there if I’m not a friend or family member or health professional’* [Doula 051]. Although almost two thirds of doulas (64 %, *n* = 45/70) said their role had never been misunderstood by a professional, 13 % (*n* = 9/70) said this had happened once and 23 % (*n* = 16/70) said this had happened more than once: ‘*treated like a lady’s mum. But once discussed, my role is understood*’ [Doula 039].

### Views on ‘matching’

The doula service staff were responsible for choosing which doula would support which woman, a process they described as ‘matching’. The doulas’ views on the importance of different factors in this process reflected the inherent subjectivity of matching. They rated ‘getting on well together’ as the most important factor (98.5 %, *n* = 67/68), but most (70 %, *n* = 47/67) rated ‘similar background’ as unimportant. ‘Speaking the same language’ and ‘doula’s age’ were both rated as unimportant by nearly half the sample (41 %, *n* = 29/70 for language and 47 %, *n* = 32/68 for age). This appears consistent with the necessarily pragmatic approach to matching and realities of doula availability and client demographics, because many doulas were not a similar age or background to the women they supported. Language support was not necessarily available. Half of the doulas (*n* = 36/71) said that they had supported a woman who did not speak English, and a substantial proportion (42 %, *n* = 30/71) had supported a non-English speaker with whom they had no common language.

The doulas were also asked whether they had experienced a match that was ‘not the best fit’ and 21 % (*n* = 15/71) said they had. In most cases this was attributed to the doula’s own inexperience at the time, or to the woman wanting something which the service did not offer (such as childcare). Only a few referred to problems with the actual ‘match’, because of personality or age difference (*‘Some people you get on better with than others’* [Doula 018]; ‘*Young woman who did not want support from an older doula’* [Doula 027]).

For some doulas, a dearth of ‘matches’ was a source of frustration:

‘*maybe we’re not getting enough mums through the doors to support, … I think I’m a wasted resource, because I’ve been a doula for two years and I’ve only supported four mums,..my main concern is that it won’t work if we don’t get consistent work, there’s no proper management of it, you get frustrated.’* [Doula 067]

### Barriers and challenges

#### Not attending birth

Over 40 % of doulas (41 %, *n* = 28/69) said they had been unable to attend a labour/birth that they had planned to attend. Reasons for this were: the woman did not contact the doula (25 %, *n* = 7/28); the doula was not available (29 %, *n* = 8/28); circumstances changed and someone else accompanied the woman (25 %, *n* = 7/28); labour occurred sooner than expected (29 %, *n* = 8/28); or a health professional did not allow it (4 %, *n* = 1/28). Most doulas said they felt ‘*disappointed*’ [Doula 003, Doula 029, Doula 090] or ‘*gutted*’ [Doula 001, Doula 017, Doula 053] at not having attended the birth, with some also feeling ‘*guilty*’ [Doula 073], ‘*very bad*’ [Doula 046] and ‘ *that I had let her down’* [Doula 001]. Others accepted the situation more philosophically when it was the mother’s choice, especially if she had support from her partner or a family member instead (‘*The lady either made the decision not to let me know or had enough support. So I’m ok with that. Happy.’* [Doula 039].

### Unable to support a woman as they would have liked

Thirty doulas had encountered a situation apart from missing labour where they were not able to support a woman as they would have liked (42 %, *n* = 30/71). In some cases, this was due to the complexity of the woman’s needs (47 %, *n* = 14/30); 40 % (*n* = 12/30) due to the boundaries of the doula service; 33 % (*n* = 10/30) due to lack of time; 17 % (*n* = 5/30) because of limits placed by health professionals. Additional reasons given were: language and cultural barriers, barriers placed by the woman’s family; the mother disengaging; and reasons personal to the doula.

### Communication difficulties

36 % (*n* = 25/70) of doulas reported communication problems with a woman (27 %, *n* = 19/70), her partner or husband (6 %, *n* = 4/70) or other family members (3 %, *n* = 2/70). These were nearly all ascribed to lack of a shared language, but some related to a husband/partner or family members wanting to maintain control over a woman or not being comfortable with the doula’s presence. In addition, 20 % of doulas (*n* = 14/70) reported communication difficulties with a health or social care professional: ‘*Being ignored by one or two midwives on the labour ward once I introduced myself as a doula. Most are lovely though.’* [Doula 069].

### Feeling out of their depth

Doulas were asked if they had ever felt out of their depth. Two (3 %) answered ‘often’; 18 (26 %) ‘occasionally’ and 49 (71 %) ‘never’. In some cases doulas who felt out of their depth related this to supporting women with complex problems (such as substance abuse, domestic abuse, the baby being removed from the mother’s care). Other doulas referred to language problems, women’s inappropriate expectations (e.g. expecting childcare for older children or housework), hostility from a midwife, or feeling they needed more knowledge (for example, of the benefits system) or skills practice (for example, massage and birth positions).

### Managing endings

The doula’s support ended six weeks after birth at four of the sites, and 12 weeks after birth at one site. The doulas’ open text responses indicated that endings could be challenging for both doulas and the women they supported. The doulas described three strategies to prepare a woman for the ending of support: being explicit from the outset about the end date , and giving reminders nearer the time; ensuring that the woman was aware of other local sources of support (for example, children’s centres); and building up the woman’s ability to cope on her own by positive reinforcement. Doulas felt that this would leave the woman ‘*prepared and empowered*’ [Doula 086], ‘*self-sufficient*’ [Doula 060] or ‘*independent…confident*’ [Doula 047], and for some this was a measure of success that made the ending easier: ‘*It’s nice to see mum standing on her own two feet and that you have done your role well’* [Doula 010]; ‘*It’s a service with a natural time line. Bit like teaching a bird to fly*’ [Doula 088].

Most doulas (71 %, *n* = 45/63) prepared something for the woman as part of the ending: 67 % of those who prepared something (*n* = 30/45) gave the woman a birth story; 62 % (*n* = 28/45) gave photographs; 29 % (*n* = 13/45) said that they prepared an account of the time spent together; and 7 % (*n* = 3/45) gave a small card or gift for the mother, the baby or both.

The main issue that doulas said made an ending difficult was when they had become friends with the woman, because according to the doula service policy they were not allowed to have further contact: *‘When you have a real friendship and both want it to continue’* [Doula 028]; ‘*Never being able to see or speak to that family again’* [Doula 086]. A second issue causing difficulties for the ending was if the mother still had unmet needs; if she had not yet been able to become established with other services; if there were no other services for her to move onto; or if she was very isolated and not yet fully ready to cope: ‘*When a mum has really needed the support and doesn’t think she could cope alone… they sometimes feel abandoned’* [Doula 066].

Some doulas said that it was easier to overcome their own feelings of loss if they went on to support another woman quickly, or sought emotional support from doula service staff: ‘*Hardest part of being a doula. It is easier if meeting a new mum that week*’ [Doula 069]. Although many doulas said they did not know how endings could be improved, some had suggestions; most commonly that the ending of support should be more flexible around the mother’s needs, or phased out gradually: ‘*6 weeks should be a guide. Sometimes a little more is needed e.g. hospital appointments for mum or baby’* [Doula 039].

Most respondents (77 %, *n* = 51/66) said they would like to have updates about the women that they had supported, and 50 % (*n* = 32/64) said that there had been women with whom they would have liked to have stayed in touch after the official ending. From the pre-specified response options, this was because of: concerns about the woman or children (22 %, *n* = 14/64); feeling that the woman needed continued contact (20 %, *n* = 13/64) or that she had no-one else (11 %, *n* = 7/64). Responses to the further option of ‘Other (please say what)’ reflected that this was due to actual or potential friendship, commented on by 15 doulas. By contrast, a few doulas thought that on-going contact would carry its own risks, which could be emotional (‘*It’s best not to follow-up otherwise the emotional connection will be difficult to break off’* [Doula 047]) or practical (‘*If we could stay in touch with the mother it may put the volunteer in difficult positions e.g. being called to a problem she is having or constant phone calls*’ [Doula 042].

Despite the doula services’ rule against on-going contact, a third of doulas (*n* = 21/64) reported contact beyond the official ending. Doula service policy forbade doulas to accept gifts from women, but two-thirds (*n* = 41/64) said that they had been offered a token of appreciation by a mother they had supported. Five (12 %) of these said that this had caused a problem: ‘*Mum became very upset when she was told by the doula office that I could not keep the gift.’* [Doula 069]

### Impact of volunteering on doulas

#### Emotional impact

The doulas were asked whether being a doula had affected them emotionally in terms of revisiting their own previous experiences of childbirth. A third of those who felt the question was applicable (30.5 %, *n* = 18/59) said that they had been affected. Most commonly they said that this had been a positive process *(‘I understand my own experiences now and the impact they have had on my life and am able to move on’* [Doula 003]), although it could also lead to feelings of disappointment (‘*Feeling sad that my ‘mum’ didn’t have a birth like I had which was empowering and amazing, where my ‘mum’ had lots of drugs and a horrible time. I thought I could help her more than I did*’ [Doula 006]).

A third of respondents (31 %, *n* = 22/70) said that being a doula had exposed them to difficult experiences that had affected them emotionally, particularly being present at a difficult birth, and the ending of the support . However, for some this exposure had had a positive impact: ‘*Positive effect as realised how strong I can be!* ’[Doula 023]; ‘*Made me feel lucky to have what I have*’ [Doula 019].

### Impact on health and wellbeing

Over half of respondents (56 %, *n* = 39/70) considered that their health and wellbeing had improved as a result of being a volunteer doula, usually because of an increased sense of confidence and self-worth, sometimes explicitly linked to improved health behaviours: ‘*More confidence - I got out more, started to build a new life for myself, cycled more too!*’ [Doula 023]. For the small number of doulas (3 %, 2/70) who reported ill effects on their health, this was mainly because of tiredness through juggling volunteering with other commitments or when supporting a woman during a long labour.

### Social and family impact

A large majority of respondents endorsed three social benefits of volunteering as a doula ‘somewhat’ or ‘a great deal’: meeting new people (99 %, *n* = 69/70), increased awareness of other people’s cultures (96 %, *n* = 67/70), and feeling more involved in the local community (87 %, *n* = 61/70). Far more doulas felt that there was a positive rather than a negative impact on their families: 83 % (*n* = 58/70) felt that their family had been affected by their increased knowledge and improved ability to access services, and 71 % (*n* = 48/68) said that their parenting knowledge and skills had increased, compared to 36 % (*n* = 25/70) who said they were less available to their family.

### Work related impacts

As reported above, the majority of doulas were to some extent motivated to volunteer by the possibility of further training or employment, and most agreed ‘somewhat’ or ‘a great deal’ that being a doula had benefited them in work-related ways. Fifty-five doulas said they had been given the opportunity to work as part of a team (81 %, *n* = 55/68), 80 % (*n* = 54/68) that they had been able to test out a maternity, health or social care work environment, 73 % (*n* = 50/68) that it had increased their confidence regarding working. There were, however, two doulas (3 %) who said that volunteering had decreased their confidence regarding working.

### Other impacts

Fifty doulas answered an open question inviting them to describe any other ways in which they had been affected by being a volunteer doula; most comments were positive. Some said they had become less judgemental: ‘*It has opened my eyes to how sheltered my life was. Also a lot of my opinions were misguided and ill-informed. I am a lot more open to many things and I do genuinely feel less judgemental in a positive way*’ [Doula 033]. Some felt that they had acquired important life skills: ‘*It has made me a better friend and given me skills to support anyone in my life*’ [Doula 069]. A third group referred to fulfilment and increased confidence: ‘*It has made me more pro-active and has encouraged me to engage with people’* [Doula 047]. For others it had helped with career choices: ‘*now a newly qualified midwife’* [Doula 023]; *‘(it) helped me to decide NOT to pursue a career in midwifery’* [Doula 051]. The small number of negative comments referred to their distress at the hardship of others’ lives, childcare commitments, or a dispiriting insight into the culture of the local health service.

### Overall satisfaction

97 % of volunteers (*n* = 69/71) said they would recommend being a doula to others and on a scale of 0-5, 69 % (*n* = 49/71) rated their experience at ‘5’ with a further 22.5 % (*n* = 16/71) rating ‘4’. However, 8.5 % (*n* = 6/71) rated below ‘4’ of whom two rated their experience as ‘0’ (these were the same doulas who would not recommend it to others and who had stopped volunteering because of dissatisfaction with service staff).

## Discussion

The volunteer doulas in this study were providing a service that is different in composition and context to many of the existing models of doula support; available comparisons on which to draw for discussion of our findings are limited. However, the experiences of volunteer doulas in this research resonate with some of the issues reported in other volunteer or peer support initiatives for childbearing women. Resistance from health professionals [22] and lack of engagement from family members [23] were reported in maternity peer support initiatives and misunderstandings about role and concerns about overlapping roles identified in peer support services in mental health services [24].

Volunteer doulas were motivated to become involved for a combination of personal and work-related reasons, consistent with the findings of the small amount of existing qualitative research on unpaid doulas [7, 15]. Most had found the training very valuable (as in Granville and Sugarman, [15]), and both the training and the volunteering fulfilled doulas’ expectations with regard to improving future education or work opportunities. Doulas cited in particular their increased confidence, the qualification gained, the opening of new work or training opportunities, the experience of team working and the chance to test out a health or social care work environment. At the same time, the doulas were almost unanimously satisfied that they were able to make a valuable contribution by supporting women during pregnancy and birth, a much higher satisfaction rating with this aspect of the role than in either of the two North American surveys of paid doulas [4, 5]. There was also evidence that doulas were a positive resource as their volunteering was building social capital [25] in the doulas’ communities–both bonds with other doulas (which half of the volunteers said was important for on-going motivation) and bridges with women from different cultural backgrounds and into the community generally. However, the small number of dissatisfied responses indicated clearly that doulas were prevented from fully enjoying their volunteering role if they did not feel well supported by the doula service staff and frustrated if they were not allocated to clients.

Most research amongst doulas has focused on their role as birth supporters, and to a lesser extent their postnatal contribution [2]. In this research slightly more doulas believed that their most valuable contribution was in the antenatal period, compared with at birth or postnatally. This should be noted by those who refer women to volunteer doula services. Whilst views varied on e.g. whether doulas should be of a similar age or background to the women supported, almost all agreed that the most important factor in a successful match was ‘getting on together’. This affords doula services flexibility in the matching process. The significant number of doulas who established a rapport with women with whom they did not share a common language is noteworthy. Future research could explore how that positive relationship is achieved when communicating through an interpreter or when language support is not available.

A large minority of doulas had encountered challenges in their role, including missing a birth that they had planned to attend, being unable to support a mother as they would have liked, communication difficulties, having their role misunderstood, or feeling out of their depth (this last point echoing the volunteer doulas in Low et al, [7]). Some had also experienced some challenges in relationships with midwives, but in contrast to the antagonism frequently reported with paid doulas [4, 26], most said relationships were positive most of the time. Difficulties had mainly been in the past before midwives understood the purpose of the volunteer doula role. Most doulas who had stopped volunteering had done so not because of any of these challenges but for personal reasons. Whether or not they had stopped volunteering, around half of the respondents said that doulas could be encouraged to continue volunteering by better recognition from the health services, more contact with other doulas, or payment. However only a third of respondents thought that doulas *should* be paid, suggesting that many saw intrinsic value in their role as volunteers. Achieving good relationships at the organisational level between statutory NHS maternity and doula services, clarity and recognition of roles appears important for the experiences of volunteer doulas.

Volunteering as a doula had had a substantial personal impact on the doulas themselves: many felt they had gained important life skills, become more confident and less judgemental, and had improved their health and wellbeing. Some found that volunteering had made them revisit their own experience of childbirth, and some had been exposed to an emotionally challenging situation during their volunteering; either scenario could have positive or negative emotional consequences. For new providers of doula services, it is important that both positive and more challenging aspects of the role are highlighted in the recruitment process. We noted that most of the services provided debriefing support for their volunteers, which doulas valued.

There was dissonance between the policy of the doula services that friendship was not part of the doula role, and the subjective experiences of some doulas that friendship could be an instrumental part of the role, or a natural human reaction within the relationship. This plurality of views on the nature of the relationship reflects findings from other volunteer peer support projects [27]. The biggest emotional issue for the volunteers was managing the ending of the relationship at 6–12 weeks after birth and the (not always observed) rule banning further contact between the woman and volunteer. This wish for a less abrupt ending reflected both the friendship that developed in many relationships and the on-going needs that several mothers had at this time. The most common suggestion for improving endings was for a phased or flexible ending more closely based on the mother’s needs. For both new and established doula services, achieving a more acceptable approach to the ending of doula support is arguably a key priority. This aspect of the service affects the experiences of a significant number of doulas and the women they support [18], with implications for emotional distress and dissatisfaction with role and service and that could undermine the value afforded by the support.

This research is the largest independent evaluation of trained volunteer doula services for vulnerable women in England, involving five sites with varied populations in different parts of the country, and 89 doulas with a range of ages, ethnic groups and levels of education. A weakness was the disappointing response rate of 34.5 %, despite reminders and assurances of confidentiality; this naturally limits generalisability. The research may have lacked relevance for former doulas. This low response rate may also have been because the research team did not have direct contact with the doulas but, for reasons of confidentiality, had to rely on the services as intermediaries, or because doulas were concerned that this research could pose a threat to the service and therefore did not engage.

## Conclusion

Training and volunteering as an unpaid doula can be a rewarding experience, in terms of both personal development and future employment prospects and the ability to test out work in health and social care. There are a number of potential learning points from this research for those planning or operating volunteer doula services. Firstly, the model of training used by these five services is both valued and effective in preparing for the role of volunteer doula. Secondly, services could consider the potential merits of more flexible endings or giving more post-ending updates to doulas, creating opportunities for doulas to be updated on families’ progress, if they and the families wish it. Thirdly, motivation to continue volunteering is highly affected by a doula’s positive sense of helping other women and feeling that they have made a difference; to avoid demotivation, services should ensure that a doula is matched with a woman as soon as possible after completing her training. Lastly, active support from service staff is fundamental to enable a doula to flourish as a volunteer, so services should be careful not to underinvest in their capacity to deliver this support.
